# Supplementary material for: Comparative genomics provides new insights into the diversity, physiology, and sexuality of the only industrially exploited tremellomycete: Phaffia rhodozyma
Source: BMC Genomics. 2016 Nov 9;17:901. doi: 10.1186/s12864-016-3244-7 (PMC5103461; doi:10.1186/s12864-016-3244-7)
Supplement: Additional file 6: — List of orphan genes with links to PFAM (related to Additional file 1: Table S1). (ZIP 1428 kb) [file 12864_2016_3244_MOESM6_ESM.zip › PFAM_UNIQUE/G03610_P.html]

#                                                                            --- full sequence --- -------------- this domain -------------   hmm coord   ali coord   env coord
  
# target name        accession   tlen query name           accession   qlen   E-value  score  bias   #  of  c-Evalue  i-Evalue  score  bias  from    to  from    to  from    to  acc description of target
  
#------------------- ---------- ----- -------------------- ---------- ----- --------- ------ ----- --- --- --------- --------- ------ ----- ----- ----- ----- ----- ----- ----- ---- ---------------------
  
G03610\_P             -            540 CgtA                 PF06306.6    347    0.0071   14.2   2.4   1   1   1.8e-06     0.011   13.6   2.4   242   325   281   368   271   387 0.78 - Beta-1,4-N-acetylgalactosaminyltransferase (CgtA)  
G03610\_P             -            540 DUF2252              PF10009.4    385       1.1    6.7   5.6   1   2     0.014        81    0.6   0.4   134   220   128   219    84   224 0.68 - Uncharacterized protein conserved in bacteria (DUF2252)  
G03610\_P             -            540 DUF2252              PF10009.4    385       1.1    6.7   5.6   2   2   6.8e-05      0.41    8.2   0.7   100   166   257   333   233   390 0.76 - Uncharacterized protein conserved in bacteria (DUF2252)  
G03610\_P             -            540 Flagellar\_rod        PF05149.7    289      0.82    7.5   4.8   1   2       0.2     4e+02   -1.4   1.2    19    81   300   320   276   344 0.51 - Paraflagellar rod protein  
G03610\_P             -            540 Flagellar\_rod        PF05149.7    289      0.82    7.5   4.8   2   2   0.00015      0.29    8.9   0.1     5    55   478   528   474   537 0.88 - Paraflagellar rod protein  
G03610\_P             -            540 FTA4                 PF13093.1    213       2.6    6.1   6.2   1   3      0.38   5.7e+02   -1.6   0.1   146   174    95   123    85   151 0.78 - Kinetochore complex Fta4 of Sim4 subunit, or CENP-50  
G03610\_P             -            540 FTA4                 PF13093.1    213       2.6    6.1   6.2   2   3   0.00018      0.27    9.3   0.4    30    82   290   339   231   423 0.75 - Kinetochore complex Fta4 of Sim4 subunit, or CENP-50  
G03610\_P             -            540 FTA4                 PF13093.1    213       2.6    6.1   6.2   3   3      0.71   1.1e+03   -2.5   0.1    12    39   473   499   453   512 0.53 - Kinetochore complex Fta4 of Sim4 subunit, or CENP-50  
G03610\_P             -            540 Kinetocho\_Slk19      PF12709.2     87     0.076   11.8   6.0   1   2    0.0042       5.1    5.9   1.0    28    81   283   332   260   339 0.77 - Central kinetochore-associated  
G03610\_P             -            540 Kinetocho\_Slk19      PF12709.2     87     0.076   11.8   6.0   2   2    0.0021       2.5    6.9   0.3    45    84   489   528   484   530 0.92 - Central kinetochore-associated  
G03610\_P             -            540 TMCO5                PF14992.1    280       2.9    5.6   9.7   1   2    0.0002      0.12   10.1   2.4    84   215   211   356   205   365 0.71 - TMCO5 family  
G03610\_P             -            540 TMCO5                PF14992.1    280       2.9    5.6   9.7   2   2       0.8   4.8e+02   -1.7   0.9   109   142   472   505   397   527 0.69 - TMCO5 family  
G03610\_P             -            540 WXG100               PF06013.7     86       2.3    7.0   5.4   1   4      0.13   2.6e+02    0.4   0.0    51    77    91   117    86   125 0.80 - Proteins of 100 residues with WXG  
G03610\_P             -            540 WXG100               PF06013.7     86       2.3    7.0   5.4   2   4      0.61   1.2e+03   -1.8   0.1    52    68   302   318   295   323 0.45 - Proteins of 100 residues with WXG  
G03610\_P             -            540 WXG100               PF06013.7     86       2.3    7.0   5.4   3   4     0.056   1.1e+02    1.6   0.0    58    78   452   472   448   479 0.82 - Proteins of 100 residues with WXG  
G03610\_P             -            540 WXG100               PF06013.7     86       2.3    7.0   5.4   4   4      0.01        21    3.9   0.1    51    81   486   516   483   518 0.89 - Proteins of 100 residues with WXG  
G03610\_P             -            540 YlqD                 PF11068.3    131    0.0052   15.5   1.4   1   4       6.9   4.2e+03   -3.6   0.1    56    75   115   134    98   153 0.49 - YlqD protein  
G03610\_P             -            540 YlqD                 PF11068.3    131    0.0052   15.5   1.4   2   4   8.6e-06    0.0052   15.5   1.4    58   105   292   339   260   347 0.89 - YlqD protein  
G03610\_P             -            540 YlqD                 PF11068.3    131    0.0052   15.5   1.4   3   4       1.7     1e+03   -1.6   0.1   115   131   372   388   366   388 0.79 - YlqD protein  
G03610\_P             -            540 YlqD                 PF11068.3    131    0.0052   15.5   1.4   4   4      0.94   5.6e+02   -0.8   0.1    67   107   490   530   431   537 0.62 - YlqD protein
